# Voluntary Restoration: Mitigation’s Silent Partner in the Quest to Reverse Coastal Wetland Loss in the USA

**DOI:** 10.3389/fmars.2019.00511

**Published:** 2019-08-28

**Authors:** Rachel K. Gittman, Christopher J. Baillie, Katie K. Arkema, Richard O. Bennett, Jeff Benoit, Seth Blitch, Julien Brun, Anthony Chatwin, Allison Colden, Alyssa Dausman, Bryan DeAngelis, Nathaniel Herold, Jessica Henkel, Rachel Houge, Ronald Howard, A. Randall Hughes, Steven B. Scyphers, Tisa Shostik, Ariana Sutton-Grier, Jonathan H. Grabowski

**Affiliations:** 1 Department of Biology and Coastal Studies Institute, East Carolina University, Greenville, NC, United States; 2 Natural Capital Project, Woods Institute for the Environment, Stanford University Stanford, CA, United States; 3 School of Environmental and Forest Sciences, University of Washington, Seattle, WA, United States; 4 Northeast Regional Office, United States Fish and Wildlife Service, Hadley, MA, United States; 5 Restore America’s Estuaries, Arlington, VA, United States; 6 The Nature Conservancy, Baton Rouge, LA, United States; 7 The National Center for Ecological Analysis and Synthesis, University of California, Santa Barbara, Santa Barbara, CA, United States; 8 National Fish and Wildlife Foundation, Washington, DC, United States; 9 Chesapeake Bay Foundation, Annapolis, MD, United States; 10 The Water Institute of the Gulf, Baton Rouge, LA, United States; 11 The Nature Conservancy, University of Rhode Island Bay Campus, Narragansett, RI, United States; 12 Office for Coastal Management, National Oceanographic and Atmospheric Administration, Charleston, SC, United States; 13 Gulf Coast Ecosystem Restoration Council, New Orleans, LA, United States; 14 United States Environmental Protection Agency Gulf of Mexico Program, Gulfport, MS, United States; 15 Gulf Coast Ecosystem Restoration Team, Natural Resource Conservation Service, United States Department of Agriculture, Madison, MS, United States; 16 Department of Marine and Environmental Sciences, Marine Science Center, Northeastern University, Nahant, MA, United States; 17 Office of Habitat Conservation, National Oceanographic and Atmospheric Administration, Silver Spring, MD, United States; 18 Earth System Science Interdisciplinary Center, University of Maryland, College Park, MD, United States

**Keywords:** marsh, conservation, coastal management, habitat loss, ecosystem function

## Abstract

Coastal ecosystems are under pressure from a vast array of anthropogenic stressors, including development and climate change, resulting in significant habitat losses globally Conservation policies are often implemented with the intent of reducing habitat loss. However, losses already incurred will require restoration if ecosystem functions and services are to be recovered. The United States has a long history of wetland loss and recognizes that averting loss requires a multi-pronged approach including mitigation for regulated activities and non-mitigation (voluntary herein) restoration. The 1989 “No Net Loss” (NNL) policy stated the Federal government’s intent that losses of wetlands would be offset by at least as many gains of wetlands. However, coastal wetlands losses result from both regulated and non-regulated activities. We examined the effectiveness of Federally funded, voluntary restoration efforts in helping avert losses of coastal wetlands by assessing: (1) What are the current and past trends in coastal wetland change in the U.S.?; and (2) How much and where are voluntary restoration efforts occurring? First, we calculated palustrine and estuarine wetland change in U.S. coastal shoreline counties using data from NOAA’s Coastal Change Analysis Program, which integrates both types of potential losses and gains. We then synthesized available data on Federally funded, voluntary restoration of coastal wetlands. We found that from 1996 to 2010, the U.S. lost 139,552 acres (~565 km^2^) of estuarine wetlands (2.5% of 1996 area) and 336,922 acres (~1,363 km^2^) of palustrine wetlands (1.4%). From 2006 to 2015, restoration of 145,442 acres (~589 km^2^) of estuarine wetlands and 154,772 acres (~626 km^2^) of palustrine wetlands occurred. Further, wetland losses and restoration were not always geographically aligned, resulting in local and regional “winners” and “losers.” While these restoration efforts have been considerable, restoration and mitigation collectively have not been able to keep pace with wetland losses; thus, reversing this trend will likely require greater investment in coastal habitat conservation and restoration efforts. We further conclude that “area restored,” the most prevalent metric used to assess progress, is inadequate, as it does not necessarily equate to restoration of functions. Assessing the effectiveness of wetland restoration not just in the U.S., but globally, will require allocation of sufficient funding for long-term monitoring of restored wetland functions, as well as implementation of standardized methods for monitoring data collection, synthesis, interpretation, and application.

## INTRODUCTION

Globally, human activities have resulted in the loss of over 70% of the habitats present in 25 identified hotspots for biodiversity (Brooks et al., 2002; Ceballos et al., 2015). Habitat loss in coastal ecosystems, in particular, has been significant, with 40% to 85% of salt marshes, seagrasses, mangroves, and oyster reefs estimated to be degraded or lost regionally and globally (Kennish, 2001; Valiela et al., 2001; Waycott et al., 2009; Beck et al., 2011). Until the latter half of the twentieth century, primary anthropogenic drivers of habitat loss, such as residential and industrial development and agriculture, continued largely unchecked in most countries. Even as the proto-environmental movement became mainstream in the 1950s and early 1960s, environmental concerns largely centered on air and water pollution as a public health issue (Dewey, 1998). Increasing public awareness of environmental issues in the 1960s and 1970s, often attributed to widely publicized environmental disasters, such as oil spills, pollution in the Great Lakes, and effects of insecticide use, birthed and rapidly advanced the modern environmental movement in the United States, Europe, and elsewhere (Dryzek et al., 2003; Dunlap and Mertig, 2014). This movement directly contributed to the enactment of several environmental policies and programs, including the U.S. Clean Air Act, U.S. Clean Water Act, U.S. Endangered Species Act, the European Union Environmental Action Programmes, and the International Union for Conservation of Nature’s Red List of Threatened Species. Collectively, these efforts have likely reduced the rates of habitat degradation for many critically valuable habitats regionally (Salzman, 1990; Arnold, 1991; Noss et al., 1997; Fischman, 2004). While the effectiveness of landmark environmental policies on air and water quality have been well-documented (e.g., Wooley and Wappett, 1982; Knopman and Smith, 1993; Lynch et al., 1996; Likens et al., 2001; Lyon and Stein, 2008), implementing laws and environmental programs aimed at mitigating or compensating for habitat destruction has been challenging. In the U.S., approaches to wetland protection, in particular, have evolved as policymakers and the public became increasingly aware of the causes and ecological consequences of wetland loss and degradation (Institute for Water Resources, 2018).

In 1987, the U.S. Environmental Protection Agency (EPA) convened the National Wetlands Policy Forum (NWPF), with a primary goal of addressing which policies should be adopted or amended to protect and conserve wetland resources (National Wetlands Policy Forum, 1988). The intent of the NWPF was to shift United States wetland regulation toward a policy of “No Net Loss” (NNL), specifically recommending that Federal legislation “establish a national wetlands protection policy to achieve no overall net loss of the nation’s remaining wetland base, as defined by acreage and function, and to restore and create wetlands, where feasible, to increase the quality and quantity of the nation’s wetland resource base” (National Wetlands Policy Forum, 1988; Bendor, 2009). The United States Army Corps of Engineers (USACE) and the EPA entered into a mitigation Memorandum of Agreement in 1990, which articulated the Clean Water Act Section 404 regulatory policy that permit applicants minimize wetland loss to the extent feasible and provide compensatory mitigation for unavoidable wetland impacts (Bendor, 2009). USACE is primarily responsible for ensuring adequate mitigation consistent with USACE and EPA regulations established under Section 404 of the Clean Water Act (Page and Wilcher, 1990; Allen and Feddema, 1996; Hough and Robertson, 2009). NNL as a policy goal, with more recent compensatory mitigation regulations, arguably continues to be a motivating force for wetlands conservation and restoration actions in the United States (Salzman and Ruhl, 2007; Bendor, 2009; Hough and Robertson, 2009).

The effectiveness of current regulations in conserving and promoting restoration of wetlands and associated ecosystem functions has been frequently questioned because of insufficient wetland mitigation following impacts, inadequate monitoring of restored wetlands to ensure recovery of function, and geographic discrepancies between where wetlands are impacted and where they are restored (Breaux and Serefiddin, 1999; Matthews and Endress, 2008; Bendor, 2009). Indeed, studies of compensatory wetland mitigation in the 1990s and early 2000s from states on the Northeast, Southeast and Pacific coastlines of the United States found less than half of permitted projects to be in compliance and approximately one in four projects did not attempt any mitigation at all (Florida Department of Environmental Regulation, 1991; DeWeese, 1994; Allen and Feddema, 1996; Brown and Veneman, 2001; Sudol and Ambrose, 2002). Further, compensatory wetlands often differ significantly in structure and function from natural reference wetlands (Balcombe et al., 2005; Spieles et al., 2006; Matthews and Endress, 2008; Hossler et al., 2011). To address these failures with compensatory mitigation, the USACE issued the “Compensatory Mitigation for Losses of Aquatic Resource Final Rule” in 2008 requiring real estate and financial instruments to protect and provide long-term stewardship of mitigation sites, respectively (Van den Bosch and Matthews, 2017). However, very few studies have addressed rates of mitigation compliance since 2008 (but see Hill et al., 2013), due in part to a major shift toward the use of in-lieu fees and mitigation banks as opposed to onsite mitigation (Hough and Harrington, 2019), and, as such, the impact of the 2008 Mitigation Rule on compliance remains unknown (Morgan and Hough, 2015).

Improved compliance with CWA Section 404 permit conditions is vital to further reduce the loss of wetland acres, to say nothing of habitat function. However, because compensation for the CWA Section 404 permitted projects is not always required or successful, voluntary restoration efforts will likely be necessary to achieve NNL. Wetland degradation and loss is also occurring as a result of natural (e.g., storm events) and anthropogenic (e.g., groundwater and oil extraction, shoreline hardening) processes that are not being prevented or mitigated under current U.S. policies and regulations (Baumann and Turner, 1990; Flournoy, 2003; Hough and Robertson, 2009; Flournoy and Fischman, 2013). Therefore, restoration efforts that compensate for wetland losses attributable to factors beyond those triggering mitigation by current U.S. laws are likely to be necessary if wetland gains are to outpace wetlands losses.

To determine how much restoration may be necessary to outpace current and future wetland losses, we first assessed how coastal wetlands have changed in the United States in the most recently reported 15 years period spanning 1996–2010, shortly after the implementation of NNL. We then synthesized data on Federally funded wetland restoration efforts that were not mandated by current U.S. compliance or mitigation requirements, termed “voluntary” herein, to determine if current efforts have the potential to outpace coastal wetland losses now and in the future. We then attempted to identify local and regional hotspots of wetland change and compare those loss/gain hotspots with restoration efforts in those areas. Further, because voluntary restoration projects were not driven by mitigation requirements that often mandate restoration to be in close spatial proximity to impacted wetlands, we investigated the degree to which siting of voluntary restoration may be creating local and regional “winners” and “losers.” Finally, we make recommendations for determining how much and where future wetland restoration should occur and what additional data should be collected to inform these decisions.

## APPROACH

### Coastal Wetland Change in the United States

To assess how coastal wetlands have changed in the United States, we reviewed the literature and publicly available datasets on wetland coverage and trends. Only recently have technological advances allowed for national-scale assessments of wetland extent and its change over time (Davidson, 2014; NOAA, 2018a). We elected to use the data available from the National Oceanographic and Atmospheric Administration (NOAA) Coastal Change Analysis Program (C-CAP) to determine coastal wetland change since 1996, the earliest available year for which C-CAP data exist for all coastal U.S. counties. NOAA C-CAP produces nationally standardized land cover and land change data for the coastal regions of the U.S (30-m pixel resolution, based on LandSAT imagery, NOAA, 2018a). Landcover classifications include intertidal areas, wetlands, and adjacent uplands. C-CAP calculates and publishes data on landcover change every 5 years, with change data currently available for the 5 years periods ending in 1996, 2001, 2006, and 2010. Change in land cover over each 5 years period is determined via comparison of land-cover imagery and classification of changes in land cover using a combination of models, ancillary data, and manual edits (McCombs et al., 2016). An accuracy assessment of the change analyses from 2006 to 2010 showed an overall accuracy in classifying land cover change ranging from 82.3 percent to 85.6 percent (McCombs et al., 2016).

For this study, we used extent and change data for palustrine and estuarine wetlands from NOAA C-CAP summarized at the coastal county level over the three, 5 years periods between 1996 and 2010, as well as the full 15 years period, cumulatively. Palustrine wetlands include all non-tidal wetlands, as well as wetlands that occur in tidal areas in which salinity due to ocean-derived salts is below 0.5 psu. Estuarine wetlands include all wetlands that occur in tidal areas in which salinity due to ocean-derived salts is equal to or greater than 0.5 psu. Palustrine wetlands and estuarine wetlands were further subdivided into three subcategories: forested, scrub-shrub, and emergent (Table 1; NOAA, 2018b). We extracted wetland extent and change data for U.S. “coastal shoreline counties,” which we define as counties that have coastlines bordering the open ocean, or contain coastal high hazard areas (V-zones, see adapted from NOAA, 2018c), to allow direct comparison to available restoration data (see Voluntary coastal wetland restoration efforts in the United States section below) compiled for the same coastal shoreline counties, referred to as coastal counties herein.

### Voluntary Coastal Wetland Restoration Efforts in the United States

We synthesized data on voluntary coastal habitat restoration projects funded by the NOAA, EPA, the U.S. Fish and Wildlife Service (USFWS), the U.S. Department of Agriculture Natural Resource Conservation Service (USDA NRCS), and the National Fish and Wildlife Foundation (NFWF) (see Table 2). Projects were cross-checked across agencies to ensure that projects funded by multiple Federal sources were not double-counted in the resulting dataset. Projects reported from each aforementioned Federal source were not solely funded by the reporting source, but were instead funded by a combination of Federal, state, and private funds via multi-entity partnerships and fund-matching requirements. Because USACE currently lacks a centralized database for voluntary restoration projects, we were unable to include those data (Vanderbilt, personal communication). Mitigation projects completed to fulfill CWA mitigation requirements or to comply with the National Resource Damage Assessment (NRDA) program were not included, as these projects are intended to mitigate or replace habitats being lost as a direct result of regulated action. We focused on voluntary restoration projects to assess the potential for these efforts to compensate for wetland losses attributable to direct human actions, as well as natural and indirect anthropogenic causes of wetland loss (e.g., storm events, sea-level rise, hydrological modification). Wetland restoration projects included in this study encompassed a wide variety of restoration techniques, including but not limited to invasive species removal, hydrologic reconnection, and wetland vegetation planting. These voluntary projects were implemented to fulfill a broad range of goals, such as improving local water quality or restoring habitat for a threatened or endangered species, depending on the mission and mandates of the Federal agency partner involved (see Table 2 for information on restoration projects data sources).

Data availability varied across sources, with restoration projects awarded from 2006 to 2015 being available from NOAA, USFWS, USDA NRCS, and EPA’s Gulf of Mexico, Chesapeake Bay and San Francisco Bay programs. Only projects awarded from 2011 to 2015 by EPA’s National Estuaries Program (NEP) and NFWF were available. The habitat type, location (coastal county), and amount restored (area, in acres), were reported for each project. We then extracted all freshwater wetland, tidal wetland, and mangrove restoration projects from this larger dataset to compare to the NOAA C-CAP data. Freshwater wetlands are defined as wetlands without salt or tidal influence, including forested, scrub-shrub and emergent wetlands. Tidal wetlands were defined as forested, scrub-shrub, and emergent vegetation subjected to tidal inundation excluding wetlands dominated by mangrove species. To allow for comparison to the NOAA C-CAP data, we reclassified restoration projects as palustrine or estuarine based on the definitions described above (Table 1).

## RESULTS

### Coastal Wetland Change (1996–2010)

In 2010, there were 5,442,458 acres (~22,025 km^2^) of estuarine wetlands and 23,230,861 acres (~94,012 km^2^) of palustrine wetlands in the 282 conterminous coastal counties of the United States (NOAA, 2018a). A majority of extant estuarine wetlands in the U.S. was emergent tidal wetlands dominated by rooted herbaceous hydrophytes (86%), with the remainder being tidal scrub-shrub (5%) and forested (9%) wetlands. In contrast, coastal palustrine wetlands are dominated by forested wetlands (61%), with scrub-shrub and emergent wetlands making up only 15 and 24%, respectively. From 1996 to 2010, U.S. coastal counties lost 139,552 acres (~565 km^2^) of estuarine wetlands (2.5% overall) and 336,922 acres (~1,363 km^2^) of palustrine wetlands (1%).

**FIGURE 1 |:**
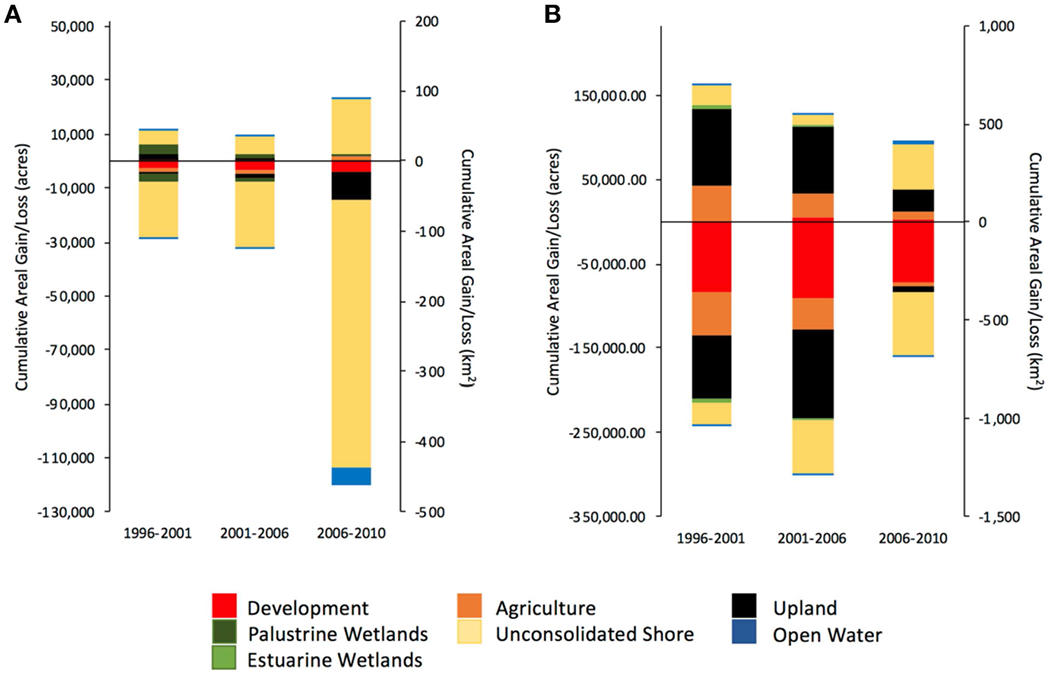
Rate of **(A)** estuarine wetland gain/loss (acres and km^2^ per year) and **(B)** palustrine wetland gain/loss in United States shoreline counties from 1996 to 2001, 2001 to 2006, and 2006 to 2010.

**FIGURE 2 |:**
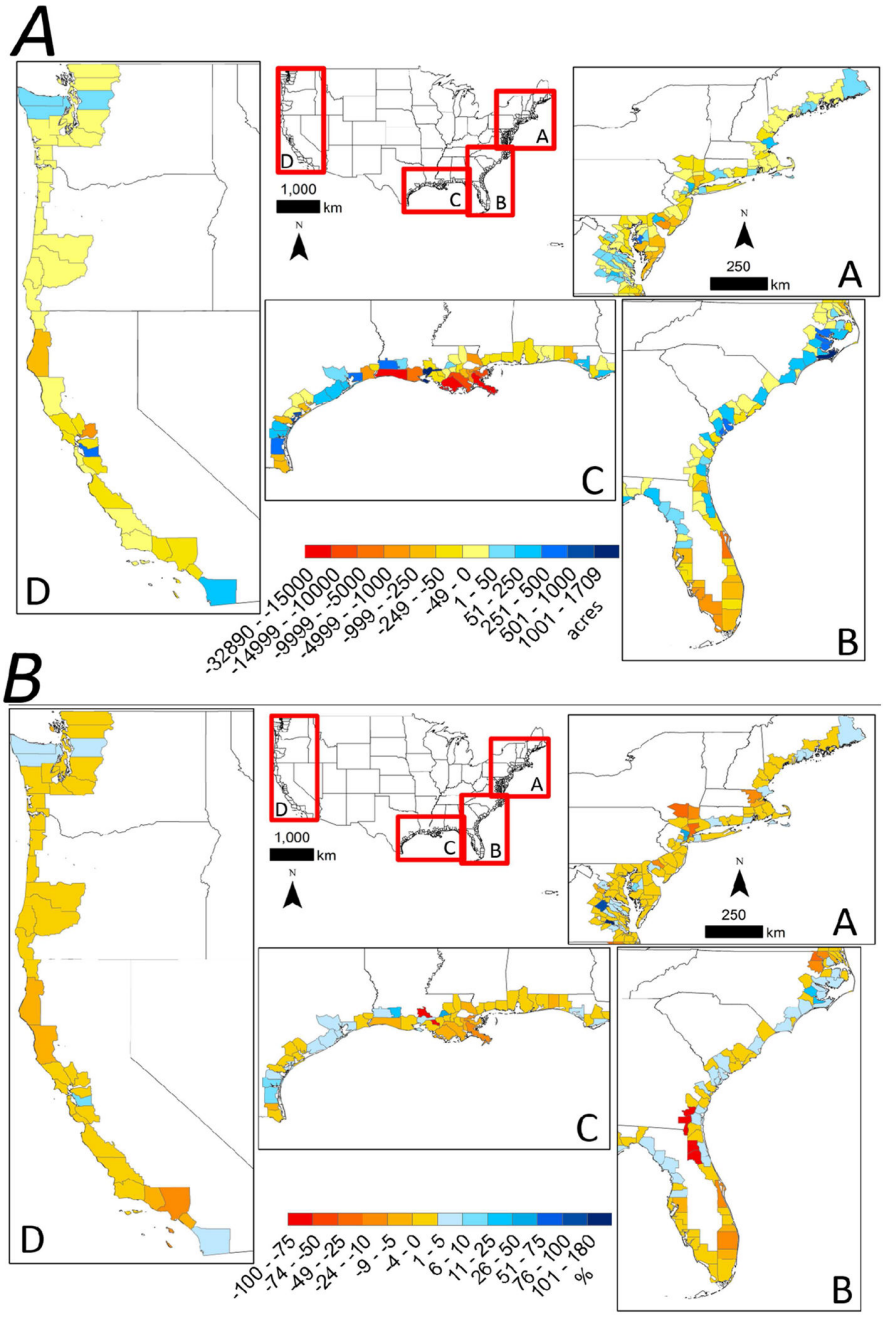
National maps with inset panels showing coastal county-level variation in the **(A)** cumulative raw acreage change in estuarine wetlands from 1996 to 2010 and **(B)** cumulative percent change in estuarine wetlands acreage from 1996 to 2010 in the (A) Northeastern coastline, (B) Southeastern coastline and gulf coast of Florida, (C) Gulf of Mexico coastline, and (D) Pacific coastline of the conterminous United States.

Twice as much estuarine wetland area was lost in the 5-year period 2001 to 2006, as compared to the previous 5 years period from 1996 to 2000 (Figure 1A). From 2006 to 2010, estuarine wetland losses were nearly nine times the losses reported from 1996 to 2001 (Figure 1A). Ninety-one percent of estuarine wetlands losses from 1996 to 2010 were attributed to losses of emergent wetlands, with conversion to unconsolidated shoreline (loose-sediment shoreline lacking vegetation) being the primary cause of loss from 1996 to 2010 (Figure 1A; see NOAA, 2018b for land-cover classification definitions). At a regional level, 87% of estuarine wetlands losses occurred in coastal counties along the Gulf of Mexico (GOM); the Northeast and Southeast Atlantic coast accounted for roughly 6% each, and Pacific coast accounted for <2% of all estuarine wetland loss (Figures 2A,B). Loss to unconsolidated shoreline was the leading cause of estuarine wetlands losses in GOM (88%), Northeast (53%), and Pacific (50%) coastal counties, while in the Southeast, loss to upland was the leading cause of estuarine wetlands loss (59%). Ninety-seven percent of estuarine wetland losses occurred in the following five states: Louisiana (80%), Florida (12%), California (2%), New Jersey (2%), and Virginia (1%) (Figure 2). Although Louisiana and Florida had the most estuarine wetlands to lose (54% of 1996 area), North Carolina, South Carolina and Georgia, states that also had considerable estuarine wetland area in 1996, all experienced estuarine wetland gains. North Carolina and South Carolina accounted for 79 and 18% of all wetland gains, respectively.

**FIGURE 3 |:**
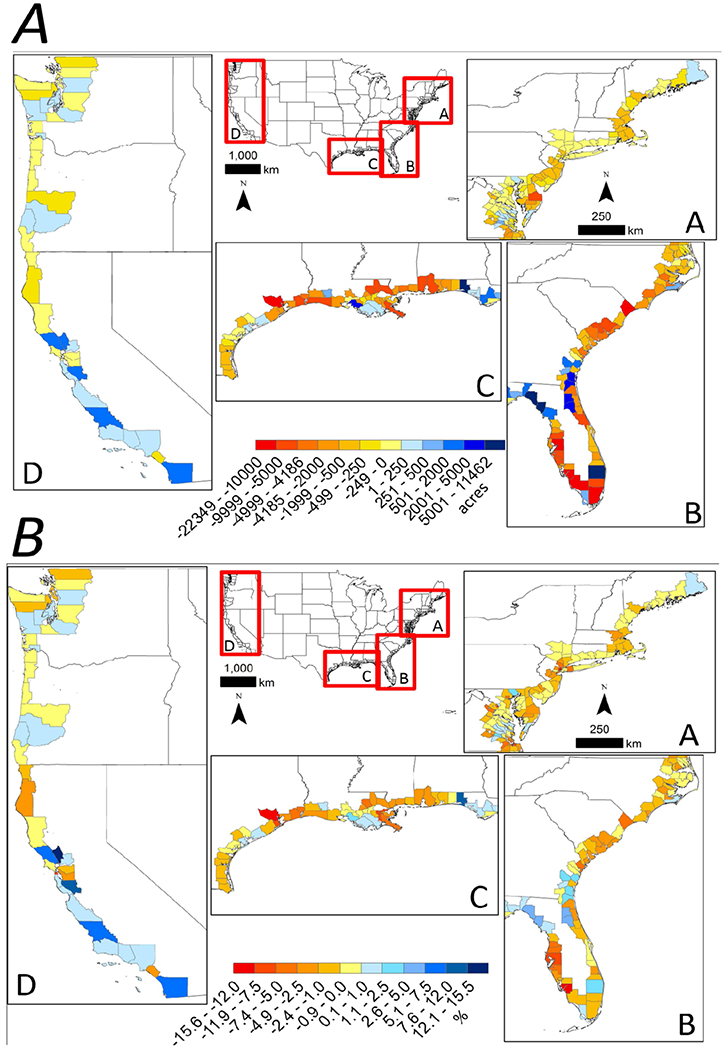
National maps with inset panels showing coastal county-level variation in the **(A)** cumulative raw acreage change in palustrine wetlands from 1996 to 2010 and **(B)** cumulative percent change in palustrine wetlands acreage from 1996 to 2010 in the (A) Northeastern coastline, (B) Southeastern coastline and gulf coast of Florida, (C) Gulf of Mexico coastline, and (D) Pacific coastline of the conterminous United States.

Palustrine wetland losses from 2001 to 2006 were nearly triple those during the previous 5 years (Figure 1B). During the period from 2006 to 2010, palustrine wetland losses had dropped to one third of those reported from 2001 to 2006 (Figure 1B). Net losses of palustrine wetlands from 1996 to 2010 represent losses of more than 1.3 million acres (~5,261 km^2^) of palustrine forested wetlands, but gains of nearly 1 million acres (~4,047 km^2^) scrub-shrub and emergent wetlands, often resulting from conversion of forested to scrub-shrub or emergent wetlands (Figure 1B). Conversion to developed lands was the greatest cause of palustrine wetland loss from 1996 to 2010 both nationally and regionally (Figure 1B). At a regional level, 52 and 34% of palustrine wetlands losses in coastal counties occurred along GOM and Southeast coastlines, respectively, while the Northeast and Pacific coastlines accounted for 13 and 1%, respectively (Figures 3A,B). Nearly 80% of palustrine wetland losses from 1996 to 2010 occurred in coastal counties within five states (listed from greatest to least loss): Florida (27%), Louisiana (17%), South Carolina (14%), Texas (12%), and North Carolina (8%, Figure 3). These states also had the most palustrine wetlands to lose in 1996 (71% of palustrine wetland area). California and the District of Columbia were the only state and Federal district to gain palustrine wetlands between 1996 and 2010, with California accounting for over 99% of those gains.

### Voluntary Coastal Wetland Restoration 2006–2015

From 2006 to 2015, the Federal government funded the voluntary restoration of 145,443 acres (~589 km^2^) of estuarine wetlands and 154,772 acres (~626 km^2^) of palustrine wetlands in U.S. coastal counties. There were 748 estuarine wetland restoration projects awarded from 2006 to 2015, with an average project size (mean ± 1 standard deviation acres) of 194 ± 1,032 acres. Similarly, there were 598 palustrine wetland restoration projects awarded from 2006 to 2015, with an average project size of 259 ± 1,221 acres. Only one estuarine and one palustrine restoration project exceeded 20,000 acres (~81 km^2^), while projects <1 acre accounted for 17% (129 projects) of estuarine wetlands restoration and 5% (29 projects) of palustrine wetlands restoration. On average, estuarine and palustrine wetlands restoration projects were completed within 2.16 ± 1.69 and 3.21 ± 2.10 years of being awarded, respectively. Restoration activities included, but were not limited to, vegetation planting, invasive species removal, prescribed burn, hydrologic reconnection, sediment stabilization/redistribution, and debris/pollutant removal.

**FIGURE 4 |:**
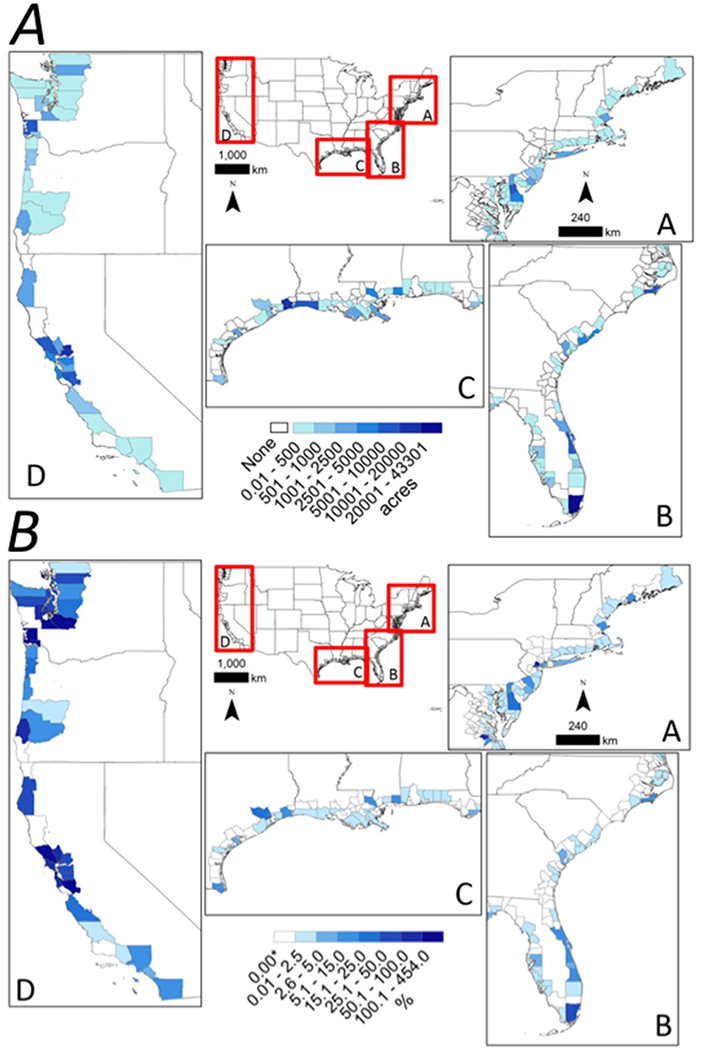
National maps with inset panels showing coastal county-level variation in voluntary estuarine wetland restoration efforts [cumulative acreage **(A)** and % of 1996 estuarine wetland area **(B)**] along the (A) Northeastern coastline, (B) Southeastern coastline and gulf coast of Florida, (C) Gulf of Mexico coastline, and (D) Pacific coastline of the conterminous United States.

More than twice as many acres of estuarine wetlands were reported as restored from 2011 to 2015 than were reported from 2006 to 2010. However, restoration projects reported by EPA NEP and NFWF accounted for 45% of the estuarine restoration that occurred between 2011 and 2015. Estuarine wetlands restoration 2011–2015 exceeded losses during each of the 5-year period between 1996 and 2010 (Figure 1A). By region, the Pacific coast accounted for 46% of estuarine wetland restoration acreage and 30% of the projects that occurred between 2006 and 2015, GOM counties accounted for 25% of acreage and 27% of projects, the Northeast accounted for 16% of acreage and 28% of projects, and the Southeast contributed 13% of acreage and 15% of projects. Seventy-five percent of the restored estuarine wetland acreage occurred in the following five states: California, Texas, Delaware, Louisiana, and Washington (Table 3; Figure 4). Restoration efforts in California accounted for more than 40% of the total area restored from 2011 to 2015 and ~40% of its 1996 estuarine wetland area (Table 3). Correlation analysis indicated a marginally significant positive relationship between cumulative estuarine wetlands loss between 1996 and 2010 and cumulative estuarine wetlands restoration between 2006 and 2015 at the state level (Spearman’s Rank Order Correlation; *p* = 0.05, rho = 0.43).

**FIGURE 5 |:**
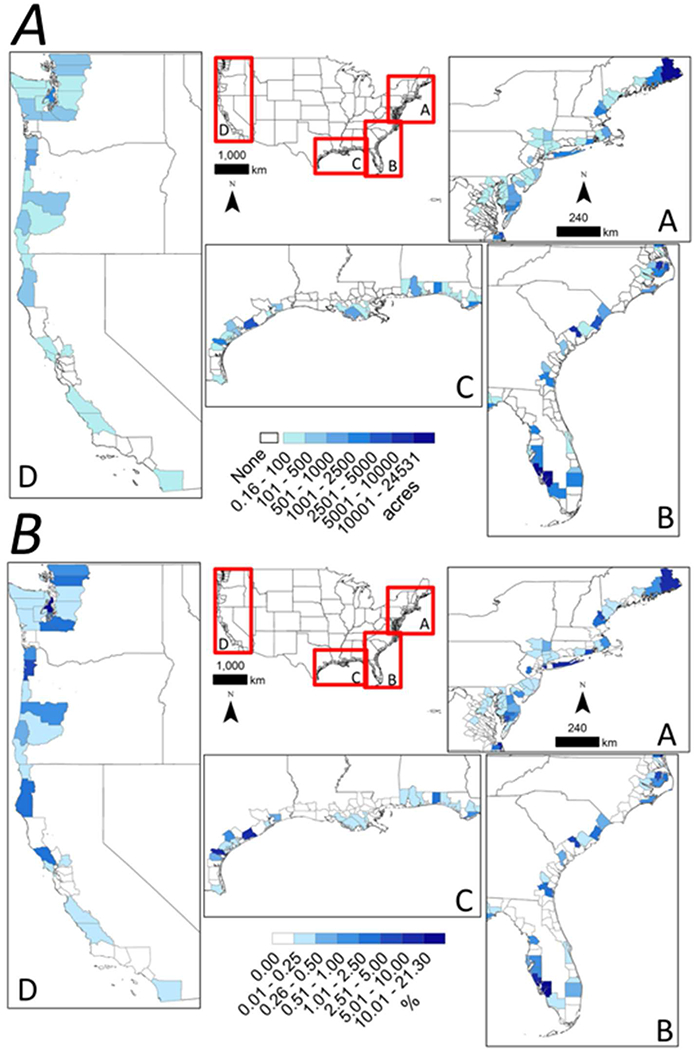
National maps with inset panels showing coastal county-level variation in voluntary palustrine wetland restoration efforts [cumulative acreage **(A)** and % of 1996 palustrine wetland area **(B)**] along the (A) Northeastern coastline, (B) Southeastern coastline and gulf coast of Florida, (C) Gulf of Mexico coastline, and (D) Pacific coastline of the conterminous United States.

The area of palustrine wetlands restored more than doubled from the first to the second half of the decade. However, palustrine restoration during the first 5-year period was less than the losses for all of the three 5-year period between 1996 and 2010 (Figure 2B). Restoration projects reported by EPA NEP and NFWF accounted for 82% of the palustrine restoration that occurred between 2011 and 2015; thus, they are responsible for all of the increase and compensate for what would otherwise have been a reduction in palustrine restoration effort from 2011 to 2015 compared to the prior 5-year period. Palustrine wetland restoration between 2011 and 2015 considerably exceeded losses from 1996 to 2001 and 2006 to 2010, but was only approximately half of the losses from 2001 to 2006 (Figure 1B). By region, coastal counties along the GOM accounted for 52% of the cumulative restored acreage of palustrine wetlands and 31% of the total number of projects that were awarded between 2006 and 2015, followed by coastal counties in the Northeast (25% of acreage, 41% of projects), the Southeast (20% of acreage, 8% of projects), and the Pacific (3% of acreage, 20% of projects). Eighty-two percent of palustrine wetland area restored from 2006 to 2015 occurred in coastal counties within five states: Florida, Maine, North Carolina, South Carolina, and Texas (Table 4; Figure 5). Restoration efforts in North Carolina accounted for ~13% of its 1996 palustrine wetland area, the highest percentage nationally (Table 4). Correlation analysis indicated a significant positive relationship between cumulative palustrine wetlands loss between 1996 and 2010 and cumulative palustrine wetlands restoration between 2006 and 2015 at the state level (Spearman’s Rank Order Correlation; *p* = 0.02, rho = 0.52).

## DISCUSSION

Coastal estuarine and palustrine wetlands continue to be lost in the United States despite significant progress in achieving the goal of NNL nationally through wetland conservation and restoration efforts (NOAA, 2010; Figure 1). Estuarine wetland restoration efforts would likely need to more than double in order to keep pace with the recent trend of estuarine wetlands losses (2006–2010; Figure 1A). This target rate of restoration assumes that all voluntary wetland restoration is creating new wetlands, as opposed to sustaining or restoring existing, but degraded, estuarine wetlands. However, several of the restoration actions reported, such as debris, pollutant, and invasive species removal, are not likely to create new wetlands and thus would not contribute to offsetting wetland losses. Similarly, despite considerable voluntary efforts, the acreage of palustrine wetlands restored was insufficient to compensate for observed losses. The future potential of voluntary estuarine and palustrine wetland restoration to help offset losses will likely depend on (1) whether restoration efforts continue at the higher rate observed in the most recent 5-year period (2011–2015), as well as (2) whether major drivers of wetland loss are halted or mitigated.

Wetland mitigation requirements are designed largely to address anthropogenically induced wetland losses attributable to direct impacts of discrete events, such as filling or draining of wetlands for development or agriculture purposes (Turner, 1997; Boesch et al., 2001; Dahl, 2011). The continued and accelerating losses of coastal wetlands due to direct human action may in part be explained by non-compliance with mitigation requirements. In the 1990s, inadequate compliance was a documented cause for continued wetland loss. For example, in California, only 33% of the 162 CWA permitted projects that were monitored were in compliance (DeWeese, 1994; Allen and Feddema, 1996; Sudol and Ambrose, 2002). Similarly, out of 391 projects requiring compensatory wetland loss mitigation projects in Massachusetts, 54% were not in compliance, 65% were smaller than required, and 22% did not attempt to conduct any mitigation effort (Brown and Veneman, 2001). Furthermore, in the early 1990s, Florida, the state with the most palustrine wetland loss over our study period, reported that out of 63 freshwater wetland mitigation permits reviewed, only four were in compliance, and 34% of projects were never constructed (Florida Department of Environmental Regulation, 1991). Acknowledging the shortcomings of permittee-responsible mitigation, the U.S. Army Corp’s 2008 Mitigation Rule incentivized the use of mitigation banks, the number of which more than doubled in the decade after the Mitigation Rule, as well as in-lieu fees (Hough and Harrington, 2019). In the wake of the 2008 financial crisis, permittee-responsible mitigation declined from 59% of compensatory measures in 2008 to 37.5% of measures in 2014 (Madsen et al., 2010; Institute for Water Resources, 2015), and the availability of funding to study mitigation sites was reduced. Thus, whether the 2008 Mitigation Rule has changed the long-term outcomes of compensatory wetland restoration or creation remains to be seen (but see Hill et al., 2013; Van den Bosch and Matthews, 2017).

While an updated evaluation of permit compliance is needed, even among completed mitigation projects, there is considerable evidence that restored wetlands do not perform ecosystem functions equivalent to those of undisturbed wetlands (Turner et al., 2001; Gutrich and Hitzhusen, 2004; Moreno-Mateos et al., 2012). Further, increased reliance on mitigation bank credits, while they in some cases may achieve greater compliance than reported in studies from the 1990s and early 2000s, is not without risks (see Levrel et al., 2017). Mitigation banking can facilitate development as opposed to avoidance (Walker et al., 2009), result in the homogenization of wetlands due to market forces (Walker et al., 2009; Dauguet, 2015), increase the spatial disconnect between impact sites and compensatory wetland creation (Ruhl and Salzman, 2006; Bendor and Riggsbee, 2011), and reduce the likelihood of long-term monitoring due to bankruptcy (Gardner and Pulley Radwan, 2005; Robertson, 2008). As such, our results revealing that 10 years of palustrine wetlands restoration efforts (2006–2015) would have more than compensated for 15 years of losses (1996–2010) had there been no loss to development highlight the need for improved monitoring of wetland mitigation projects and increased scrutiny of unregulated impacts of development on wetlands. These changes will be particularly critical to palustrine wetland conservation as predicted increases in coastal population density will likely result in continued development of coastal lands (NOAA, 2015).

Although wetland losses in many areas may be attributed to discrete or direct human actions, such as draining or filling for development agriculture, there are numerous natural and anthropogenic factors that can indirectly contribute to wetland losses. Secondary outcomes of increasing conversion to unconsolidated shore and open water are caused by natural processes, such as storms and flooding, as well as human activities, such as groundwater and oil extraction, installation of hydrologic barriers, sediment restriction, dredging, and climate change (Baumann and Turner, 1990; Turner, 1997; Brinson and Malvárez, 2002; Zedler and Kercher, 2005; Gittman et al., 2015; NOAA, 2018a). A major weakness of current U.S. habitat protection policies is that they are poorly suited to address indirect causes of wetland loss (Flournoy and Fischman, 2013). Thus, the degree to which wetland losses can be outpaced may depend on voluntary wetland restoration efforts.

Given that funding for wetland restoration in the U.S. and elsewhere is likely to remain limited in the future, policymakers and restoration practitioners must avoid wetland loss and prioritize where and how to regain lost coastal wetlands. Our results suggest that there are mismatches between regions where wetlands are being lost and where restoration efforts are occurring, with the greatest mismatch occurring in Louisiana, where considerably more wetlands are being lost than restored (Tables 3, 4). However, in 2012 and updated in 2017, Louisiana adopted a Coastal Master Plan intended to direct resources to reverse the State’s wetland loss over the next 50 years (CPRA, 2017). Further, this mismatch may be diminished in the coming decades, as billions of dollars have been allocated to habitat restoration in the GOM coast as a result of settlement dollars from the Deepwater Horizon oil spill in 2010 (Diamond et al., 2014). Efforts to rectify spatial mismatches between wetland loss and restoration will potentially enhance the efficacy of restoration and minimize future disparities that will likely occur (Elliott et al., 2019).

There is mounting evidence that anthropogenic climate change effects will not be uniformly distributed along coastlines throughout the U.S. (Weston, 2014; Schuerch et al., 2018). As climate change results in sea level rise and increased storm frequency and intensity, rates of estuarine and palustrine wetlands losses are likely to accelerate, particularly in areas with highly developed uplands and sediment deficits that prevent wetlands from either transgressing landward (i.e., coastal squeeze) or accreting fast enough to keep pace with sea-level rise (Boesch et al., 2001; Scavia et al., 2002; Nicholls and Lowe, 2004; Pontee, 2013; Weston, 2014; Peteet et al., 2018; Schuerch et al., 2018). Additionally, accelerating rates of sea level rise and associated saltwater intrusion will likely result in conversion of palustrine wetlands to estuarine wetlands, unconsolidated shore, or open water, resulting in further losses (Sallenger et al., 2012; Neubauer, 2013; Peterson and Li, 2015; Valle-Levinson et al., 2017). Thus, dedicating restoration resources to areas experiencing the greatest losses may be suboptimal if local conditions make successful restoration unlikely to be achieved and sustained. As such, allocating restoration funding to wetland construction projects in regions where human activities have negatively impacted the ecogeomorphic feedbacks that support marsh stability (e.g., flood control levees in Louisiana, canal creation in Florida) may be futile without first removing the underlying indirect causes of wetland instability (Day et al., 2005; Kirwan et al., 2010; Kirwan and Megonigal, 2013; Weston, 2014; Temmerman and Kirwan, 2015).

Interpretation of our results must be caveated with an acknowledgment that our dataset did not capture all voluntary wetland restoration (e.g., EPA NEP, and NFWF projects between 2006 and 2010, USACE state agency and NGO projects without Federal-funding partners). As stated previously, not all voluntary wetland restoration is creating new wetlands, as several of the restoration actions reported (e.g., debris, pollutant, and invasive species removal) are not creating new wetlands. Further, successful wetland restoration projects can require upwards of a decade to vegetate (Zedler and Callaway, 1999; Zedler and Kercher, 2005; Kusler, 2012), resulting in a lag in the spectral change required for detection via the remote sensing approach (Chapple and Dronova, 2017) used by NOAA C-CAP to calculate wetland change (NOAA, 2018a). Thus, the potential lag in detectability of both compensatory and voluntary wetland restoration may result in an over or under estimation of wetland losses and gains for decades or even longer. While beyond the scope of the present study, advancements in the remote sensing of fine-scale land cover changes will likely become an increasingly important tool used to inform the outcome of wetland restoration, both compensatory and voluntary, at a national scale. Despite these data limitations, the vast majority of voluntary restoration projects included in this study were awarded to state agencies and NGOs or included state or NGO partners who generally contribute a minimum of 1:1 matching funds or services. This suggests that the Federal government is a primary catalyst for funding sources of coastal wetland restoration, and that the Federal government is likely involved in much of the voluntary restoration occurring in U.S.

We recommend the following actions for improving wetland conservation and restoration in the U.S. and globally:

- Where possible, prioritize voluntary restoration efforts in the areas that have experienced the greatest losses, while also considering local and regional natural and anthropogenic factors that may influence long-term wetland restoration success;
- Establish uniform performance metrics and monitoring protocols for assessing ecosystem functions of restored wetlands;
- Ensure adequate funding for post-restoration monitoring of created and enhanced wetlands; and
- Adopt uniform reporting practices for wetland restoration projects across restoration funders and practitioners.

Most restoration projects do not have funding for long-term monitoring post-restoration (Sutton-Grier et al., 2018); thus, long-term assessments of restored wetland resilience are rare (but see Craft et al., 2008). Further, “area restored” is the most consistently reported metric for restoration projects, yet this metric provides no information on the success of restoring ecological functions and associated services. Without the ability to determine the degree to which restored wetlands are recovering ecosystem functions equivalent to those of undisturbed wetlands, restoration cannot be completely relied upon as an effective approach to wetland protection and conservation. Policymakers and practitioners should look to recent efforts to standardize monitoring of oyster reef restoration (Brumbaugh et al., 2006;Baggett et al., 2015), as well as evaluations of restored wetland ecosystem functions (Meli et al., 2014), for further guidance. In conclusion, the results of this study suggest that reversing coastal wetland losses will be challenging to achieve as climate change exacerbates wetland loss. However, given the magnitude of recent restoration efforts, it is clear that significantly increased funding and appropriate planning and siting of coastal wetland restoration has the potential to ensure that coastal wetlands and their associated ecosystem services are protected and sustained in the future.

## Figures and Tables

**TABLE 1 |:** NOAA C-CAP wetland classifications.

	Definition
**PALUSTRINE WETLANDS**	

Palustrine Forested Wetland	Includes tidal and non-tidal wetlands dominated by woody vegetation ≥5 m in height, and all such wetlands that occur in tidal areas in which salinity due to ocean-derived salts is below 0.5%. Total vegetation coverage is >20%.
Palustrine Scrub/Shrub Wetland	Includes tidal and non-tidal wetlands dominated by woody vegetation <5 m in height, and all such wetlands that occur in tidal areas in which salinity due to ocean-derived salts is below 0.5%. Total vegetation coverage is >20%. Species present could be true shrubs, young trees and shrubs, or trees that are small or stunted due to environmental conditions.
Palustrine Emergent Wetland (Persistent)	Includes tidal and non-tidal wetlands dominated by persistent emergent vascular plants, emergent mosses or lichens, and all such wetlands that occur in tidal areas in which salinity due to ocean-derived salts is below 0.5%. Total vegetation cover is >80%. Plants generally remain standing until the next growing season.

**ESTUARINE WETLANDS**	

Estuarine Forested Wetland	Includes tidal wetlands dominated by woody vegetation ≥5 m in height, and all such wetlands that occur in tidal areas in which salinity due to ocean-derived salts is equal to or greater than 0.5%. Total vegetation coverage is >20%.
Estuarine Scrub/Shrub Wetland	Includes tidal wetlands dominated by woody vegetation <5 m in height, and all such wetlands that occur in tidal areas in which salinity due to ocean-derived salts is equal to or greater than 0.5%. Total vegetation coverage is greater than 20%.
Estuarine Emergent Wetland	Includes all tidal wetlands dominated by erect, rooted, herbaceous hydrophytes (excluding mosses and lichens). These wetlands occur in tidal areas in which salinity due to ocean-derived salts is equal to or greater than 0.5% and are present for most of the growing season in most years. Total vegetation cover is >80%. Perennial plants usually dominate these wetlands.

**TABLE 2 |:** | SNAPP restoration data sources.

Data source	Year span	URL
NOAA Restoration Center	2006–2015	https://www.fisheries.noaa.gov/topic/habitat-conservation#how-we-restore
NOAA Pacific Coast Salmon Recovery Fund	2006–2015	https://www.webapps.nwfsc.noaa.gov/
EPA National Estuary Program	2011–2015	https://www.epa.gov/nep
EPA Gulf of Mexico Program	2006–2015	https://www.epa.gov/gulfofmexico
EPA San Francisco Bay Water Quality Improvement Fund	2006–2015	https://www.epa.gov/sfbay-delta/san-francisco-bay-water-quality-improvement-fund
EPA Chesapeake Bay Program	2006–2015	https://www.epa.gov/aboutepa/about-chesapeake-bay-program-office
National Fish and Wildlife Foundation	2011–2015	https://www.nfwf.org/Pages/default.aspx
USDA Natural Resources Conservation Service	2006–2015	https://www.nrcs.usda.gov/wps/portal/nrcs/site/national/home/
USFWS Fish and Aquatic Conservation Program	2006–2015	https://www.fws.gov/fisheries/
USFWS Partners for Fish and Wildlife Program	2006–2015	https://www.fws.gov/partners/
USFWS Coastal Program	2006–2015	https://www.fws.gov/coastal/
USFWS National Wildlife Refuge System	2006–2015	https://www.fws.gov/refuges/
USFWS Wildlife and Sport Fish Restoration Program	2006–2015	https://wsfrprograms.fws.gov/

**TABLE 3 |:** Voluntary estuarine restoration efforts from 2006 to 2015.

	Projects		Acres		Square kilometers		Total restoration		1996 Wetland area	
State	2006–2010	2011–2015	2006–2010	2011–2015	2006–2010	2011–2015	2006–2010	2011–2015	2006–2010	2011–2015
AL	1	1	4	1	<1	<1	<1%	<1%	<1%	<1%
CA	39	44	7,180	44,166	29	179	17%	43%	6%	40%
CT	3	18	59	97	<1	<1	<1%	<1%	<1%	1%
DE	1	18	1,980	12,003	8	49	5%	12%	3%	16%
FL	47	80	4,208	8,107	17	33	10%	8%	<1%	1%
GA	2	3	100	51	<1	<1	<1%	<1%	<1%	<1%
LA	34	31	8,732	4,707	35	19	21%	5%	<1%	<1%
MA	14	17	238	1,160	1	5	1%	1%	1%	5%
MD	25	22	118	317	<1	1	<1%	<1%	<1%	<1%
ME	5	5	247	65	1	<1	1%	<1%	<1%	<1%
MS	7	5	1,666	1,829	7	7	4%	2%	3%	3%
NC	9	12	4	5,063	<1	20	<1%	5%	<1%	2%
NH	4	1	126	<1	1	<1	<1%	<1%	2%	0%
NJ	5	16	203	3,564	1	14	<1%	3%	<1%	2%
NY	9	18	577	600	2	2	1%	1%	1%	1%
OR	14	20	1,293	1,542	5	6	3%	1%	10%	12%
RI	5	2	48	112	<1	<1	<1%	<1%	1%	1%
SC	18	9	3,578	252	14	1	9%	<1%	1%	<1%
TX	40	15	6,818	9,783	28	40	16%	9%	1%	2%
VA	8	15	52	1,470	<1	6	<1%	1%	<1%	1%
WA	53	53	4,629	8,695	19	35	11%	8%	24%	44%

The number of projects, acres, and square kilometers restored, as well as the percentage of the total national wetland area restored and the percentage of the 1996 wetland area restored are reported for coastal shoreline counties in each U.S. state for 2006–2010 and for 2011–2015.

**TABLE 4 |:** Voluntary palustrine restoration efforts from 2006 to 2015.

	Projects		Acres		Square kilometers		Total restoration		1996 Wetland area	
State	2006–2010	2011–2015	2006–2010	2011–2015	2006–2010	2011–2015	2006–2010	2011–2015	2006–2010	2011–2015
AL	2	12	144	562	1	2	<1%	1%	0%	0%
CA	13	4	466	26	2	<1	1%	<1%	<1%	<1%
CT	0	2	0	2	0	<1	0%	<1%	0%	<1%
DE	25	5	1,239	94	5	<1	3%	<1%	1%	<1%
FL	32	118	4,589	69,856	19	283	10%	66%	0%	1%
GA	5	4	1,879	1,434	8	6	4%	1%	<1%	<1%
LA	3	9	97	635	<1	3	<1%	1%	<1%	<1%
MA	15	6	322	398	1	2	1%	<1%	<1%	<1%
MD	22	22	3,702	581	15	2	8%	1%	1%	<1%
ME	7	7	13,841	5,851	56	24	29%	5%	4%	2%
MS	0	0	0	0	0	0	0%	0%	0%	0%
NC	1	14	380	12,608	2	51	1%	12%	<1%	13%
NH	65	13	1,906	290	8	1	4%	<1%	<1%	<1%
NJ	5	12	754	166	3	1	2%	<1%	<1%	<1%
NY	21	5	1,174	100	5	<1	2%	<1%	<1%	<1%
OR	30	29	409	990	2	4	1%	1%	<1%	<1%
RI	1	0	1,426	0	6	0	3%	0%	2%	0%
SC	8	5	9,601	2,887	39	12	20%	3%	1%	<1%
TX	8	12	1,419	5,543	6	22	3%	5%	<1%	<1%
VA	4	6	1,772	4,254	7	17	4%	4%	<1%	<1%
WA	34	12	3,003	373	12	2	6%	0%	1%	<1%

The number of projects, acres, and square kilometers restored, as well as the percentage of the total national wetland area restored and the percentage of the 1996 wetland area restored are reported for coastal shoreline counties in each U.S. state for 2006–2010 and for 2011–2015.
